# PTPRZ1 regulates calmodulin phosphorylation and tumor progression in small-cell lung carcinoma

**DOI:** 10.1186/1471-2407-12-537

**Published:** 2012-11-21

**Authors:** Hideki Makinoshima, Genichiro Ishii, Motohiro Kojima, Satoshi Fujii, Youichi Higuchi, Takeshi Kuwata, Atsushi Ochiai

**Affiliations:** 1 Pathology Division, Research Center for Innovative Oncology, National Cancer Center Hospital East, 6-5-1 Kashiwanoha, Kashiwa, Chiba, 277-8577, Japan; 2 Research Resident, Foundation for Promotion of Cancer Research, Chuuou-ku 5-1-1 Tsukiji, Tokyo, 104-0045, Japan; 3 Laboratory of Cancer Biology, Department of Integrated Biosciences, Graduate School of Frontier Sciences, The University of Tokyo, Kashiwa, Chiba, Japan

**Keywords:** Small cell lung carcinoma (SCLC), Protein tyrosine phosphatase (PTP), Protein tyrosine phosphatase receptor Z1 (PTPRZ1), NETs (Neuroendocrine tumors), Pleiotrophin (PTN), Calmodulin (CaM)

## Abstract

**Background:**

Small-cell lung carcinoma (SCLC) is a neuroendocrine tumor subtype and comprises approximately 15% of lung cancers. Because SCLC is still a disease with a poor prognosis and limited treatment options, there is an urgent need to develop targeted molecular agents for this disease.

**Methods:**

We screened 20 cell lines from a variety of pathological phenotypes established from different organs by RT-PCR. Paraffin-embedded tissue from 252 primary tumors was examined for PTPRZ1 expression using immunohistochemistry. shRNA mediated *PTPRZ1* down-regulation was used to study impact on tyrosine phosphorylation and *in vivo* tumor progression in SCLC cell lines.

**Results:**

Here we show that PTPRZ1, a member of the protein tyrosine- phosphatase receptor (PTPR) family, is highly expressed in SCLC cell lines and specifically exists in human neuroendocrine tumor (NET) tissues. We also demonstrate that binding of the ligand of PTPRZ1, pleiotrophin (PTN), activates the PTN/PTPRZ1 signaling pathway to induce tyrosine phosphorylation of calmodulin (CaM) in SCLC cells, suggesting that PTPRZ1 is a regulator of tyrosine phosphorylation in SCLC cells. Furthermore, we found that PTPRZ1 actually has an important oncogenic role in tumor progression in the murine xenograft model.

**Conclusion:**

PTPRZ1 was highly expressed in human NET tissues and PTPRZ1 is an oncogenic tyrosine phosphatase in SCLCs. These results imply that a new signaling pathway involving PTPRZ1 could be a feasible target for treatment of NETs.

## Background

Neuroendocrine tumors (NETs) that includes small cell lung carcinomas (SCLC), large cell neuroendocrine carcinomas (LCNEC), pancreatic neuroendocrine tumors (PanNET), medullary thyroid carcinomas (MTC), pheochromocytomas, paragangliomas, and carcinoids [1-4]. As one of the most malignant NETs, SCLC comprises approximately 15% of lung cancer cases, and basic and clinical research efforts have translated little innovation in the treatment of this disease over the past 30 years [5]. Although SCLC appears to be effectively controlled with first line chemotherapy because of its relative high sensitivity to chemotherapy and radiotherapy, most patients ultimately relapse and salvage chemotherapy is considered [6]. To identify novel drug targets against SCLC, a greater understanding of the pathology of SCLC through molecular analysis is urgently needed.

Dissection of the signaling pathways that may be involved in the regulation of SCLC growth, for example via phosphorylation or dephosphorylation of critical proteins, may shed light on new approaches for tumor elimination. Protein tyrosine phosphorylation is tightly regulated by protein tyrosine-kinases (PTKs) and protein tyrosine-phosphatases (PTPs) [7,8]. PTPs play an important role in the inhibition and control of growth as tumor suppressors, since aberrant tyrosine phosphorylation is a characteristic feature of cancer cells [7-9]. Indeed, PTPs expressed as cell surface receptors (PTPRs) have been reported to be inactivated by genetic mutations in human cancer [9,10]. On the other hand, there is mounting evidence suggesting that several PTPRs also have oncogenic function [9].

PTPRZ1, as a member of the PTPR family, is a single-pass type I membrane protein with two cytoplasmic tyrosine phosphatase domains (D1 and D2), an alpha-carbonic anhydrase domain (CA), chondroitin sulfate proteoglycans (CS-PGs) and a fibronectin type-III domain (FNIII) [11]. PTPRZ1 interacts with its ligand pleiotrophin (PTN), which is a secreted growth factor involved in angiogenesis and tumor growth [12,13]. Upon binding, PTN inactivates the phosphatase activity of PTPRZ1, which leads to an increased tyrosine phosphorylation status of important signaling molecules such as β-catenin, Fyn and RhoGAP [14-18]. With regard to cancer, PTPRZ1 expression was dramatically induced by genetic amplification caused by chronic oxidative stress and hypoxic stress through HIF-2 alpha [14,19] and several previous studies suggested that PTPRZ1 regulates cancer cell growth and cell migration [18,20-24].

In this paper, we found that PTPRZ1 is highly expressed in SCLC cell lines and specifically exists in human NET tissues. We hypothesized that PTPRZ1 functions to regulate tyrosine phosphorylation in SCLC cells and has an important role for SCLC tumor progression. To test this idea, we investigated the ability of PTPRZ1 to regulate tyrosine phosphorylation and tumor progression using SCLC cell lines.

## Methods

### Cell cultures

LN229 (glioblastoma, ATCC#CRL-2611), U87MG (glioblastoma/astrocytoma, ATCC#HTB-14), Hela (cervix ADCA, ATCC#CCL-2), Caco2 (colorectal ADCA, ATCC#HTB-37), DLD1 (colorectal ADCA, ATCC#CCL-221), HCT116 (colorectal ADCA, ATCC#CCL-247), SW480 (colorectal ADCA, ATCC#CCL-228), A549 (lung ADCA, ATCC#CCL-185), LNCaP (prostate ADCA, ATCC#CRL-1740), MCF7 (breast ADCA, ATCC#HTB-22), A431 (squamous cell carcinoma, ATCC#CRL-1555), NCI-H69 (SCLC, ATCC#HTB-119), NCI-H82 (SCLC, ATCC#HTB-175), NCI-H345 (SCLC, ATCC#HTB-180), NCI-H446 (SCLC, ATCC#HTB-171), NCI-H510A (SCLC, ATCC#HTB-184), NCI-H1436 (SCLC, ATCC#CRL-5871), and NCI-H1930 (SCLC, ATCC#CRL-5906) were originally purchased from ATCC and stocked in our Research Center. TE1 (esophagus squamous cell carcinoma), TE3 (esophagus squamous cell carcinoma), TE4 (esophagus squamous cell carcinoma), TE5 (esophagus squamous cell carcinoma), and TE10 (esophagus squamous cell carcinoma) were gifts from Dr. Sasaki (National Cancer Center Research Institute). SBC-3 (SCLC, #JCRB0818) was obtained from the JCRB and stocked in our Research Center. All cell lines were cultured in cell culture dishes (BD Biosciences) at 37°C and 5% carbon dioxide using RPMI 1640 (SIGMA), DMEM (SIGMA) supplemented with 10% fetal bovine serum (FBS, Nichirei Bioscience), or HITES Medium [25,26] supplemented with penicillin/streptomycin (Invitrogen). For the PTN assay, 100 ng/ml of recombinant human pleiotrophin/PTN (R&D Systems #252-PL) was used.

### Human cancer samples

Samples were obtained with informed consent from each individual, and the study was approved by the Ethics Committee of the National Cancer Center East Hospital. During the period from January 1992 to December 2010, a total of 252 patients who had primary tumors were treated at the National Cancer Center Hospital East, Chiba, Japan. All primary cancers with a pathologic diagnosis based on the classification schema of the WHO classification were reviewed, with 105 cases as adenocarcinoma (ADC), 61 as squamous cell carcinoma (SQCC) and 86 as neuroendocrine tumors (NETs). We used tissue microarray (TMA) to measure PTPRZ1 expression within lung tumors [27]. Each case in which more than 80% of the cancer cells reacted positively for an antibody to PTPRZ1 was recorded as positive.

### Antibodies

Antibodies used included anti-PTPRZ1 (SIGMA #015103) [28], anti-Phosphotyrosine, clone 4 G10 (Millipore #05-321), anti-Calmodulin (Santa Cruz sc-5537, Millipore #05-173, abcam ab45689), anti-phospho-Calmodulin (Santa Cruz Biotechnology sc-23760-R, Millipore #09-295) and anti-β-tublin (Cell Signaling #2146).

### Immunohistochemistry (IHC)

All immunohistochemical (IHC) analyses were performed on paraffin-embedded tissues obtained from the primary tumor in the surgical specimen. For all IHC analyses the surgically resected specimens were fixed in 10% formalin and embedded in paraffin for routine pathological examination. We prepared and used 5-μm-thick paraffin sections cut from a paraffin block containing histological findings that were representative of the tumor. The procedure for IHC was previously described [27,28]. Antigen retrieval was performed in citrate buffer solution (pH 6.0). Endogenous peroxidase was blocked with 0.3% H_2_O_2_ in methanol for 15 min and all slides were heated to 95°C by exposure to microwave irradiation for 20 min and then cooled at room temperature (RT). Slides were washed in PBS and after a 1 h incubation at RT with the primary antibodies, the slides were incubated for 30 min with a labeled polymer EnVision TM+, Peroxidase–conjugated anti-Mouse or Rabbit (Dako, Tokyo, Japan). The chromogen used was 2% 3, 3^′^-diaminobenzidine (DAB) in 50 mM Tris-buffer (pH 7.6) containing 0.3% hydrogen.

### RNA isolation and real-time RT–PCR

Cells were washed with PBS and total RNA from the cell lines was isolated with TRIzol Reagent (Invitrogen). Complementary DNA (cDNA) was synthesized using the PrimeScript® RT reagent Kit (TaKaRa, Japan). Real-time RT–PCR was carried out with specific primers and a Smart Cycler (Cepheid, Sunnyvale, CA, USA). Real-time fluorescence monitoring of the PCR products was performed with SYBR Green I fluorescent dye (TaKaRa). The levels of expression of specific genes are reported as ratios to the level of expression of *GAPDH* in the same master reaction. Synthesized primers were purchased from TaKaRa Bio with Primer Set ID given as *PTPRZ1*, 3^′^ (HA082543). GAPDH was used for normalization as control and the relative quantitation value compared to the calibrator for that target is expressed as 2^-(Ct-Cc)^.

### Western blot

Western blotting was performed as described [29]. After lentivirus infection with the vector for sh*LUC* or sh*PTPRZ1*, total cell lysate was prepared from cells cultured in complete medium. Primary antibodies were used at 1:1000 dilution and β-tubulin was used as loading control.

### Expression of short hairpin RNA (shRNA)

Plasmid construction was carried out with Gateway system (Invitrogen) according to the manufacturer’s instructions. Cloning vectors were pDNOR221 (Invitrogen) and pENTR/U6 (Invitrogen). The lentiviruses were produced using 293FT cells (Invitrogen) transfected with pCAG-HIVgp, pCMV-VSV-G-RSV-Rev, and a lentivirus vector based on CSII-CMV-RfA-IRES2-Venus (Dr. Miyoshi, RIKEN BioResource Center) expressing shRNA with the sequence described below. Transfection was achieved using Lipofectamine 2000 reagent (Invitrogen) according to the manufacturer’s instructions. Lentivirus-containing medium was filtered through a 0.45 μm filter and used for transduction of target cells. The sequences and plasmid names were; sh*LUC*: GTGCGCTGCTGGTGCCAAC (pGL3, firefly luciferase), sh*PTPRZ1*_1: GCCTATAAATTGTGAGAGCTT (pHMA017), sh*PTPRZ1*_2: GCTGCTTTAGATCCATTCATA (pHMA019), and sh*PTPRZ1*_3: GGATGGCAAACTGACTGAT (pHMA022).

### Flow cytometry

Cells were incubated with anti-PTPRZ1 antibody (SIGMA) and excess antibody was removed by washing with PBS containing 2% FBS. Polyclonal goat anti-rabbit immunoglobulin conjugated to Phycoerythrin (PE) (Jackson) was added as a secondary antibody. The cells were then washed with PBS and flow cytometric analysis was performed using a FACSCalibur and FACSAria (BD Biosciences).

### Animal studies

All of experimental *SCID* mice were handled in accordance with institutional guidelines established by the Animal Care Committee of the National Cancer Center East Hospital. H69 and H1930 SCLC cells expressing shRNA were injected into the subcutaneous tissue of SCID mice (7–8 weeks of age, CLEA, Tokyo, Japan). Tumor volume was calculated as the product of a scaling factor of 0.52 and the tumor length, width, and height were measured every week. For IHC analysis, organs were obtained from mice at 5 or 8 weeks after injection and fixed in 10% formalin.

### Statistical methods

Standard Student’s *t*-test was used to determine the significance between non-targeting control and sh*PTPRZ1* experiments. Statistical correlation was carried out using χ2test for independence (2 × 2 *contingency table*). *P* < 0.05 was considered statistically significant.

## Results

### PTPRZ1 is highly expressed in SCLC cell lines

**Figure 1:**
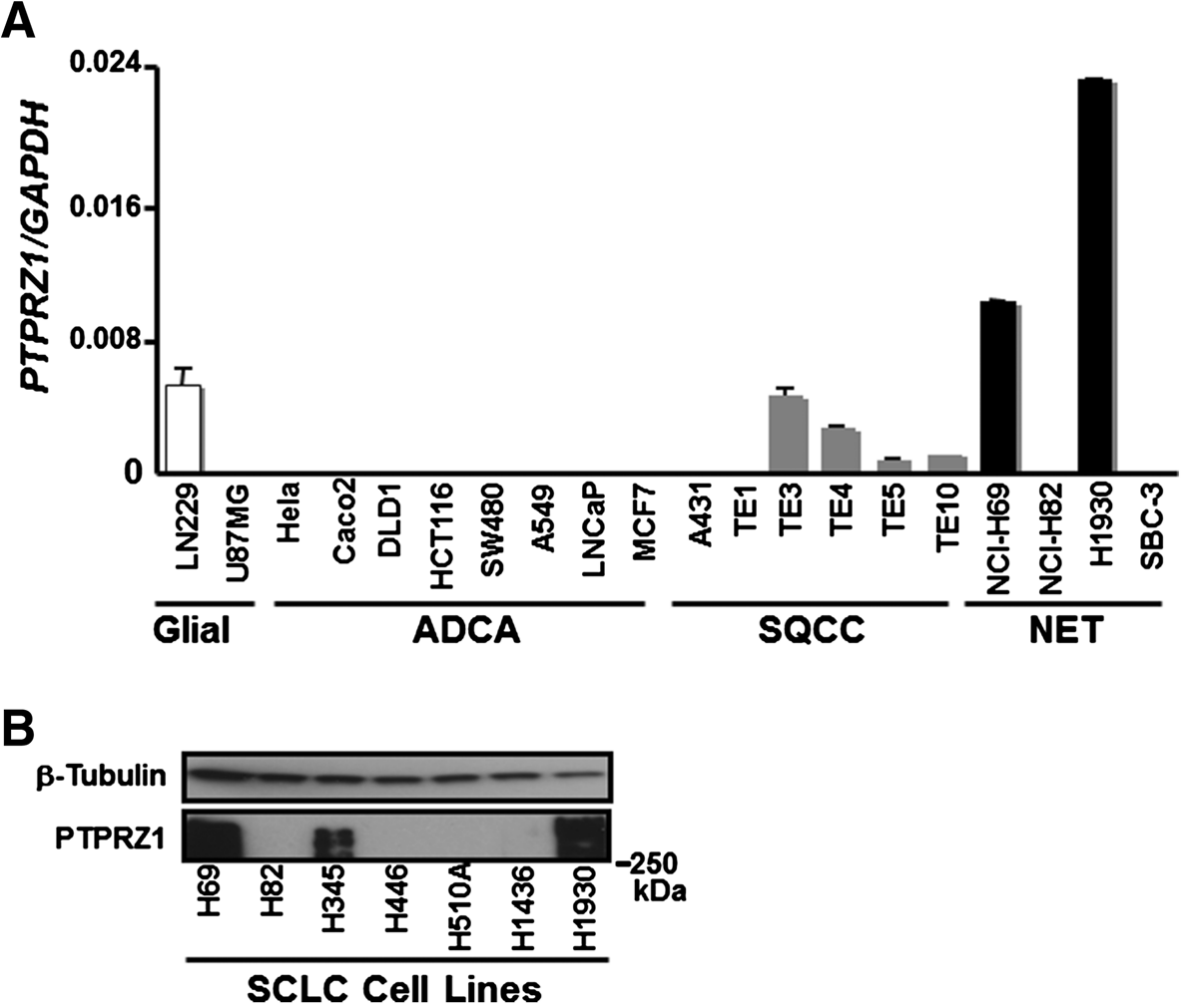
**Gene expression of PTPRZ1 among the different cancer cell lines.** Glial, adenocarcinoma (ADCA), squamous cell carcinoma (SQCC) and neuroendocrine tumor (NET) cell lines were screened for *PTPRZ1* mRNA levels (normalized to GAPDH), with standard deviation error bars shown (**A**). PTPRZ1 was expressed in SCLC cell lines at protein level (**B**).

To assess mRNA expression of *PTPRZ1* comprehensively in human cancers, we screened 20 cell lines from a variety of pathological phenotypes established from different organs by RT-PCR. We observed that two SCLC cell lines at the first screening, NCI-H69 (H69) and NCI-H1930 (H1930), expressed *PTPRZ1* mRNA at significantly higher levels than other cell lines (Figure 1A). To confirm the specificity of PTPRZ1 expression in SCLC cells, we measured PTPRZ1 protein levels by Western blotting (Figure 1B). The human *PTPRZ1* gene encodes a core protein consisting of 2315 amino acids (NCBI Reference Sequence: NP_002842) with a predicted molecular weight (M.W.) of 400 kDa, [30]. Indeed, we detected a specific band of PTPRZ1 protein at approximately 400 kDa by WB, only within SCLC cell lines expressing *PTPRZ1* mRNA at high levels (Figure 1B).

### PTPRZ1 is specifically expressed in human NET tissues

**Figure 2:**
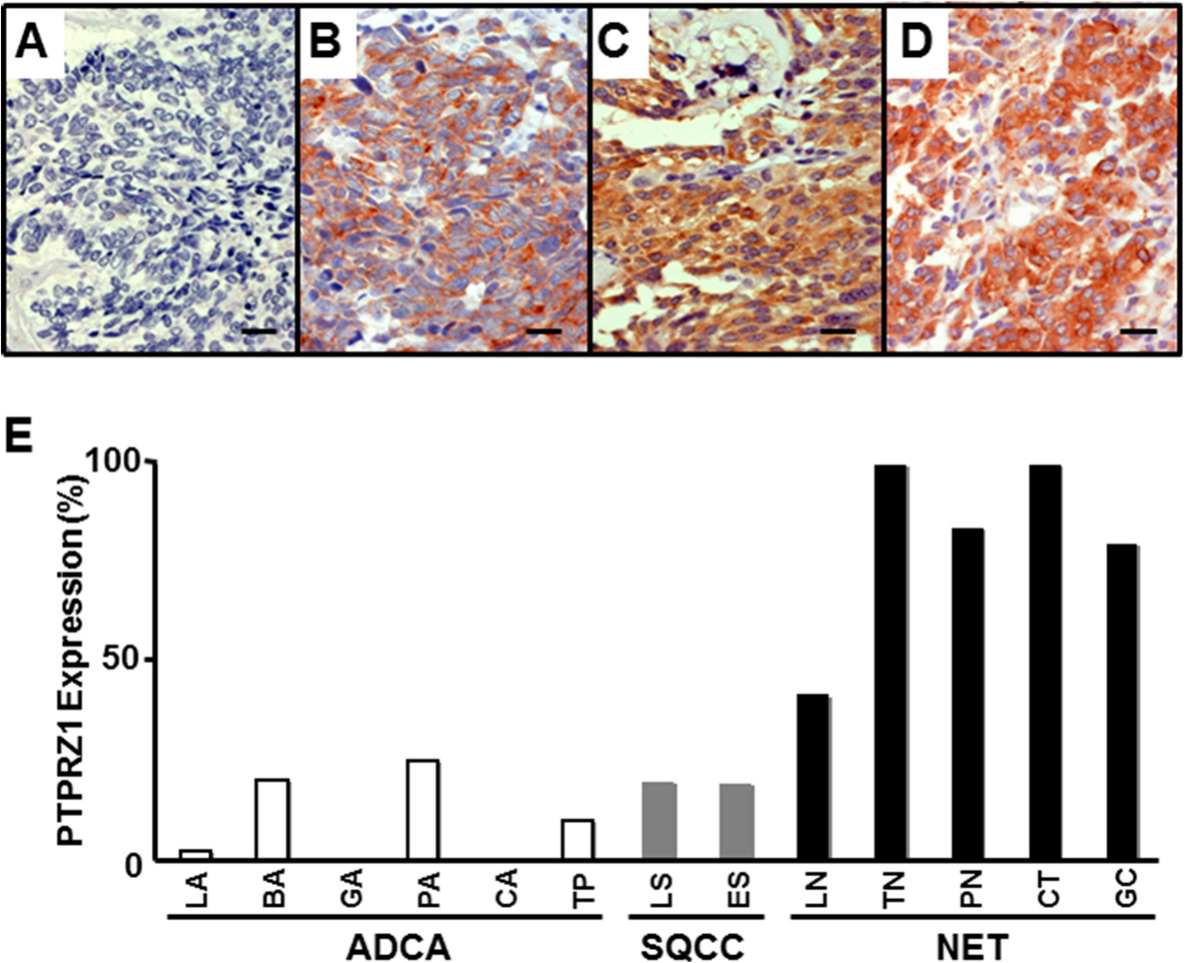
**PTPRZ1 was expressed in human NETs. A**–**D**, Representative microscopic images for PTPRZ1-negative SCLC (**A**), PTPRZ1-positive SCLC (**B**), Thyroid Medullary carcinoma (TMC) (**C**) and Pancreatic endocrine tumor (PanNET) (**D**). Scale bars are 20 μm (Magnification X40). **E**, The percentage of PTPRZ1 expression in human tumor tissues was measured in ADCA, SQCC, and NET pathological types including lung ADCA (LA), breast ADCA (BA), gastric ADCA (GA), pancreatic ADCA (PA), colon ADCA (CA), Thyroid ADCA (TA), lung SQCC (LS), esophagous SQCC (ES),lung NET (LN), thyroid NET (TN), pancreatic NET (PN), chromaffin-cell NET (CT), and gastrointestinal carcinoid (GC).

To determine globally which human tumor tissues expressed PTPRZ1, we analyzed immunohistochemical (IHC) evaluations of a variety of tumors including 105 cases of adenocarcinoma (ADCA), 61 cases squamous cell carcinoma (SQCC) and 86 cases NET. In non-tumor tissues, we specifically observed PTPRZ1 expression in the neural cells and endocrine cells such as peripheral nerves, pancreatic islets and adrenal chromaffin cells. Representative IHC evaluations of PTPRZ1-positivity (PTPRZ1+) with anti-PTPRZ1 antibody in a variety of NETs are shown in Figure 2, for PTPRZ1-negative (PTPRZ1-) SCLC (A), PTPRZ1+ SCLC (B), MTC (C), and PanNET (D). PTPRZ1 was mainly localized in the cell membrane as well as the cytosol. We found that PTPRZ1 was detected at high frequency and intensity in a variety of human NETs including 60% of SCLCs (Figure 2E, Table 1). PTPRZ1 was expressed at much higher levels in NETs (79%) than in ADCA (9%) and SQCC (20%) (Figure 2E).

**Table 1:** The IHC analysis of PTPRZ1 expression in human tumor tissues

**Tumors**	**PTPRZ1−**	**PTPRZ1+**	**%**
**Adenocarcinoma (ADCA)**			**9**
Lung ADCA	44	1	2
Breast ductal ADCA	8	2	20
Gastric ADCA	10	0	0
Pancreatic ductal ADCA	15	5	25
Colon ADCA	10	0	0
Thyroid papillary carcinoma	9	1	10
**Squamous cell carcinoma (SQCC)**			**20**
Lung SQCC	41	10	20
Esophagus SQCC	8	2	20
**Neuroendocrine tumor (NET)**			**79**
Lung NET	11	8	42
Medullary thyroid carcinoma	0	16	100
Pancreatic NET	5	26	84
Chromaffin cell tumor	0	10	100
Gastointestinal carcinoid	8	2	80

### RNAi knockdown of PTPRZ1 in SCLC cell lines

**Figure 3:**
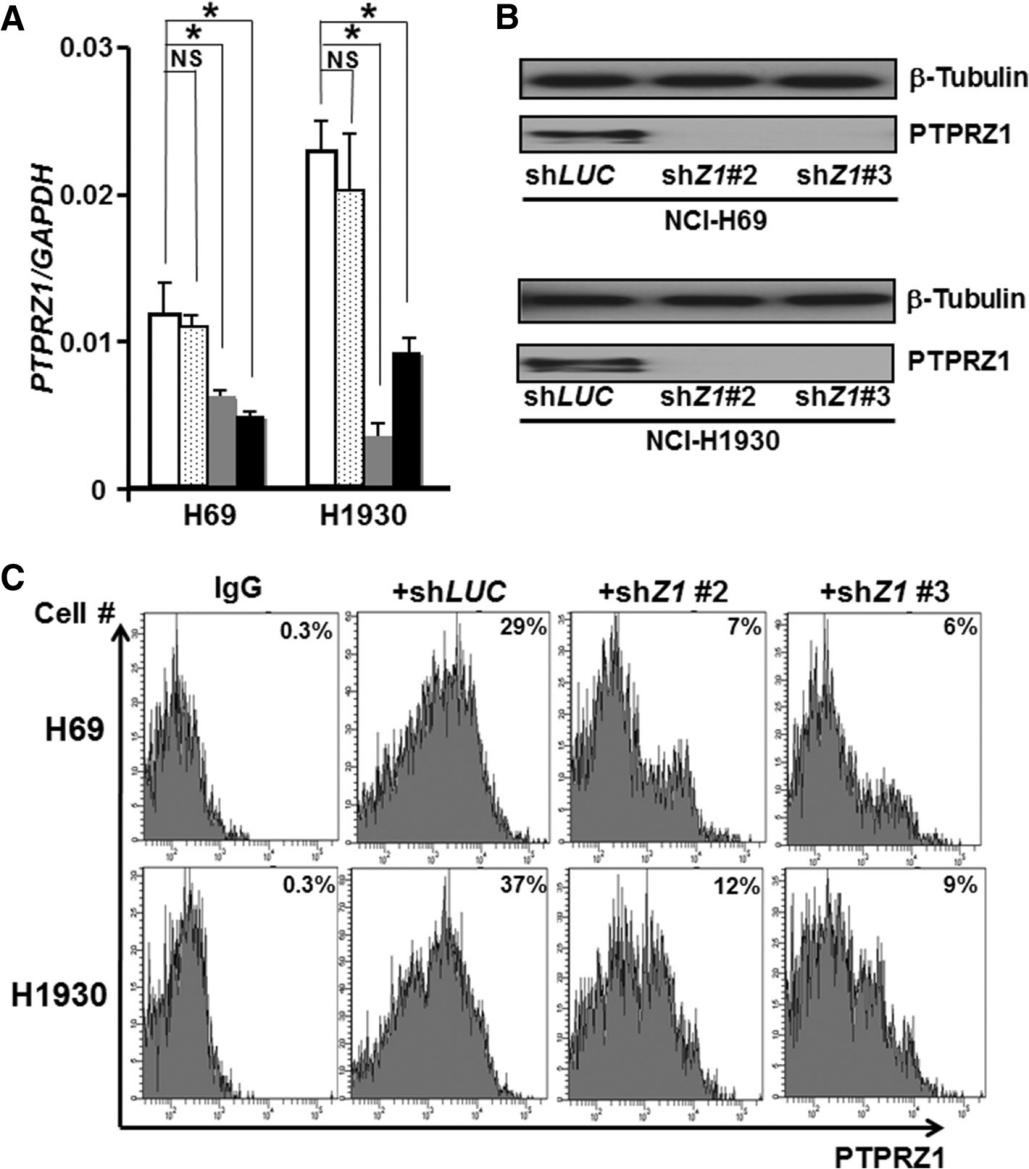
**PTPRZ1 expression was downregulated by shRNA in SCLC cell lines. A**, shRNA targeting *PTPRZ1* (sh*Z1*) successfully knock-downed *PTPRZ1* mRNA by up to 75% in the SCLC cell lines, H69 and H1930, as compared to negative control construct expressing sh*LUC*. White bars = sh*LUC*, dotted bars = sh*Z1*#1, gray bars = sh*Z1*#2, black bars = sh*Z1*#3. Error bars represent SD. Asterisk denotes *P* < 0.05 using Student’s *t* test, while NS denotes non-significant change. **B**, PTPRZ1 downregulation in H69 and H1930 was confirmed by Western blot, using β-tubulin as control for protein levels. **C**, FACS analysis of surface PTPRZ1 protein expression on H69 and H1930 cells, with sh*Z1* down-regulating PTPRZ1 expression levels.

To characterize further the function of PTPRZ1 in SCLC cells, we employed a genetic approach to repress *PTPRZ1* expression using by RNA interference (RNAi). For potential off-target shRNA effects, three different sequences of shRNA directed against *PTPRZ1* (sh*Z1*#1, #2 and #3) and a nontargeting shRNA (sh*LUC*) were constructed. While the introduction of the first construct sh*Z1*#1 in SCLC cells did not appear to down-regulate PTPRZ1 mRNA levels as compared to control sh*LUC* when measured by quantitative RT-PCR, significant reduction in mRNA expression of 75% using sh*Z1*#2 and 60% using sh*Z1*#3 could be observed in the expression of *PTPRZ1* in the SCLC cell lines H69 and H1930 (Figure 3A). WB analyses also revealed significant decreases in PTPRZ1 protein expression upon introduction of sh*Z1*#2 and #3, as compared to a control vector, in H69 and H1930 under normal culture conditions (Figure 3B). To measure cell surface PTPRZ1 levels in sh*Z1*-transduced SCLC cells, we used flow cytometry (FACS). FACS analysis of sh*LUC*-SCLC cells and sh*Z1*-SCLC cells also revealed significant reduction of PTPRZ1 expression on SCLC cellular surface from 29% to 6–7% in H69 cells and 37% to 9–12% in H1930 cells (Figure 3C).

### PTN induced calmodulin tyrosine phosphorylation in SCLC cells

**Figure 4:**
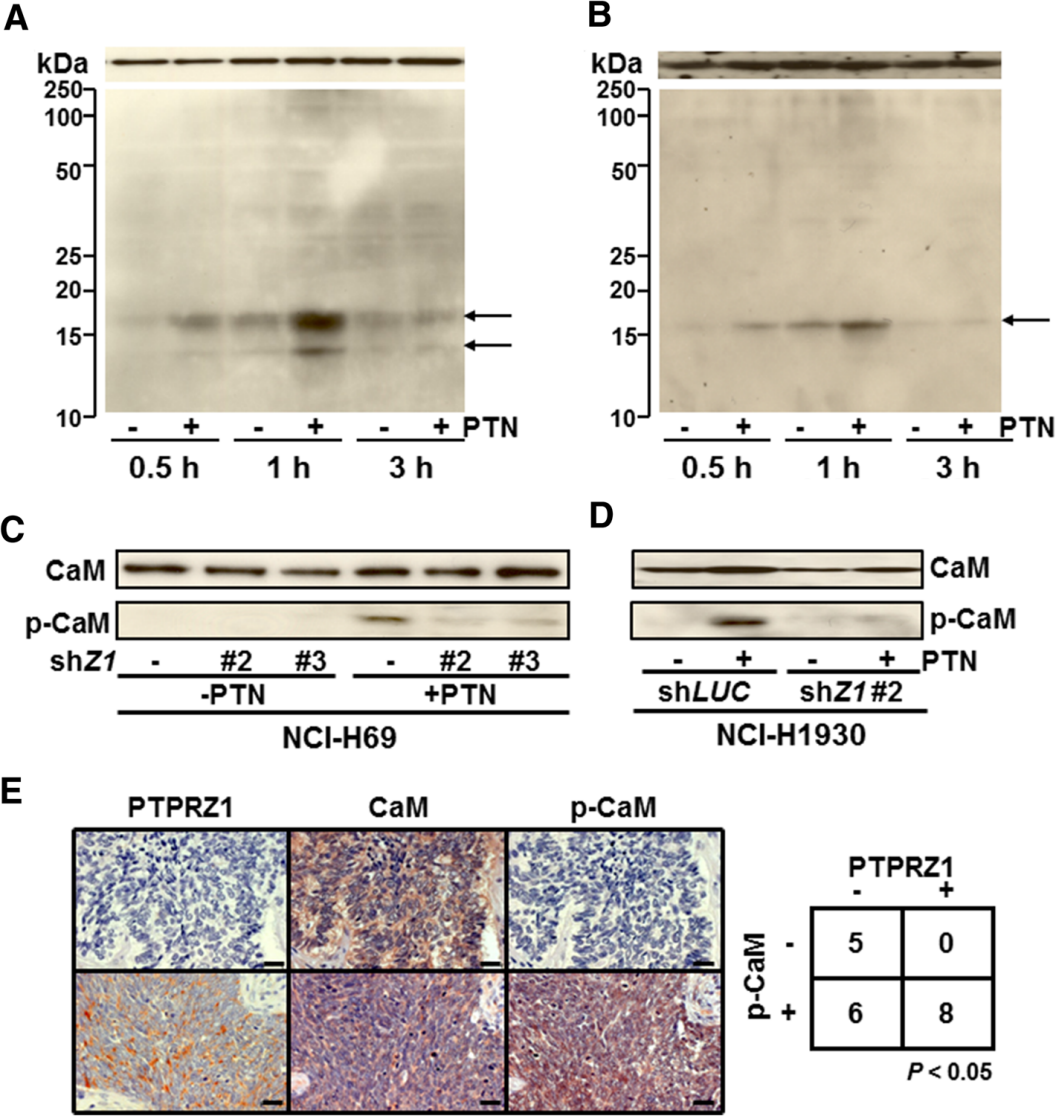
**PTPRZ1 regulates tyrosine phosphorylation in SCLC cells. A**, Tyrosine phosphorylated proteins in H69 cells were detected by Western blot with anti-pTyr antibody (4 G10) with β-catenin as loading control on top. **B**, Tyrosine phosphorylated CaM was detected by Western blot with anti-phosphorylated CaM, with total CaM on top as loading control in H69 cells. **C** and **D**, sh*Z1*s block PTN-stimulated tyrosine phosphorylation of CaM in H69 (**C**) and H1930 (**D**). PTPRZ1 promotes the phosphorylation of calmodulin in response to PTN. **E**, Correlated expression of PTPRZ1 and tyrosine phosphphorylated CaM (pCaM) in human lung NET tissues. Representative negative (−) or positive (+) images were shown. Scale bars were 20 μm (Magnification X40). *P* < 0.05 was considered statistically significant.

Although our findings demonstrated that PTPRZ1 was specifically up-regulated in SCLC cells, no studies to date have suggested a functional role for PTPRZ1 in SCLC cells. As PTPRZ1 has been linked to protein tyrosine phosphatase activity, we first assessed the ability of PTPRZ1 to regulate tyrosine phosphorylation in the response to the ligand of PTPRZ1, PTN. PTN binding to the extracellular portion of PTPRZ1 brings two molecules into close proximity and consequently the phosphatase domains dimerize in a head-to-toe arrangement with the D2 domain of one molecule blocking the active site (D1) of the second molecule, leading to suppression of phosphatase activity [31,32]. To identify molecular targets regulated by PTPRZ1 in response to PTN, we assessed tyrosine-phosphorylated proteins using an anti-phosphotyrosine antibody by WB. Interestingly, we detected two specific bands that migrated just above and below 15 kDa within 30–60 min after PTN addition to SCLC cells (Figure 4A). Although it appears that those bands could be detected at low levels in the absence of PTN, the addition of PTN significantly induced phosphorylation that peaked at 1 h. Since calmodulins (CaM) are highly abundant, 17 kDa proteins in the mammalian brain, nervous and endocrine systems and directly interact with the intracellular domain of PTPR members [33,34], we hypothesized that PTPRZ1 may normally dephosphorylate the phosphorylated tyrosine residue at Tyr99 of CaM. To test this idea, we assessed the phosphorylation of CaM using an anti- phospho-Tyr99-CaM (p-CaM) Ab and determined that the upper band could indeed be identified as CaM (Figure 4B).

### PTPRZ1 is required for the tyrosine phosphorylation of CaM induced by PTN

To verify that the addition of PTN facilitated CaM phosphorylation specifically through its receptor PTPRZ1, we utilized the H69 and H1930 cell lines in which sh*Z1* was used to knock down PTPRZ1 expression. Although PTN induced tyrosine phosphorylation of CaM in H69 cells that expressed the control sh*LUC*, the ablation of PTPRZ impaired PTN-induced CaM tyrosine phosphorylation in cells that expressed either of the two sh*Z1* constructs (Figure 4C). We confirm that PTPRZ1 was indispensable for the PTN-induced p-CaM in H1930 cells (Figure 4D).

As the serum levels of PTN were elevated in most SCLC patients in comparison to healthy controls [12], we thought PTPRZ1 expression might be correlate with the expression of phosphorylated CaM. To assess whether PTPRZ1-CaM regulation also occurred *in vivo* in human tissue, we stained for CaM and p-CaM. Indeed we found that the expression of PTPRZ1 and p-CaM was statistically correlated in human lung NET tissues (Figure 4E). These data thus demonstrate that ablation of PTPRZ1 prevents PTN-stimulated tyrosine phosphorylation of CaM in PTN-stimulated SCLC cells; the data indicate that endogenous PTPRZ1 is required for PTN-stimulating tyrosine phosphorylation of CaM in SCLC cells.

### **PTPRZ1 regulates tumor progression of SCLC in xenograft mode**l

**Figure 5:**
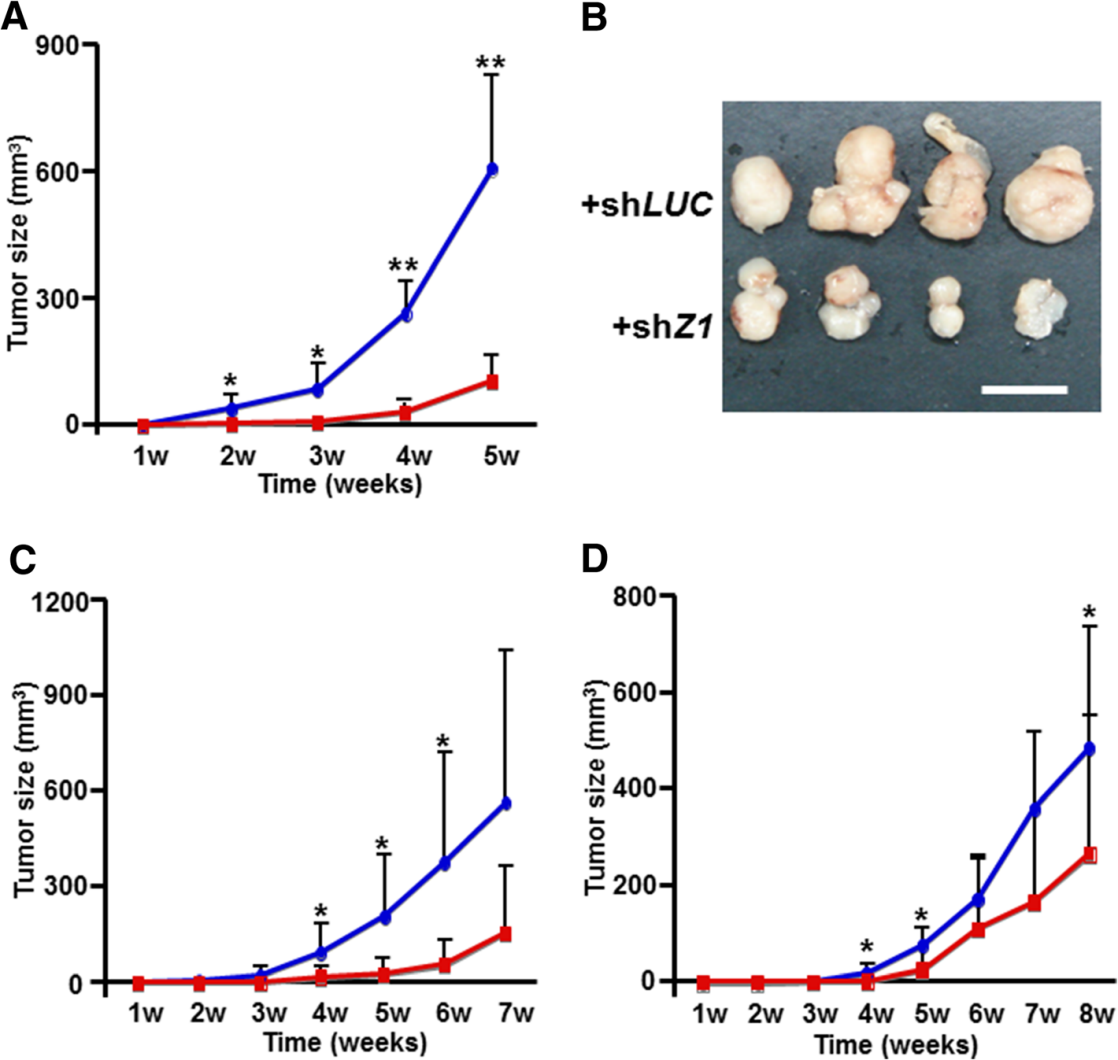
**PTPRZ1 regulates tumor progression in a SCLC xenograft mouse model. A** and **B**, *in vivo* growth of SCLC NCI-H69 expressing sh*LUC* (blue diamonds) and sh*Z1*#2 (red squares) in *SCID* mice. The loss of *PTPRZ1* in cells expressing shZ1#2 inhibited tumor growth as compared to cells expressing the control sh*LUC*. Tumor size (mm^3^) over period (**A**) and gross tumor pathology 5 weeks post-transplant are shown (**B**). Scale bar indicates 10 mm. **C** and **D**, Deficient *in vivo* growth of tumors was confirmed using H69 cells expressing sh*Z1*#3 (**C**) and H1930 cells expressing sh*Z1*#2. Control SCLC cells with shLUC are shown as blue diamonds while cells with shZ1 are shown as red squares. Error bars are SD. Asterisk, *P* < 0.05; double asterisk, *P* < 0.01; both using Student’s *t* test.

Many PTPRs play an important role as tumor suppressors [9], yet PTPRZ1 has a role in cell migration and tumor growth *in vivo* in glioma studies [20]. To determine whether overexpressed PTPRZ1 acts as a tumor suppressor or tumor promoter in human NETs, we used the severe combined immunodeficiency (*SCID*) murine xenograft model subcutaneously transplanted with human SCLC cells. 2 x 10^6^ H69 cells expressing either sh*LUC* (H69 + sh*LUC*) as a control or sh*Z1*#2 (H69 + sh*Z1*#2) were subcutaneously transplanted into the flanks of *SCID* mice (n = 7) and tumor size was measured over time. In this mouse model, H69 + sh*LUC* cells started to grow exponentially at 7 days post-transplant and progressively form tumor masses for 5 weeks (Figure 5A). In contrast, the H69 + sh*Z1*#2 cells were impaired for tumor formation until 3 weeks post-transplant such that tumors were barely recognized under the skin and were about 3-fold smaller than those in H69 + sh*LUC* cells (Figure 5A and B). Gross examination of H69 + sh*Z1*#2 tumors revealed a dramatic loss of SCLC pathology in the tumor (Figure 5B). To exclude the possibility of off-target effects of shZ2#2, we subcutaneously transplanted H69 cells expressing either sh*LUC* (H69 + sh*LUC*) as a control or sh*Z1*#3 (H69 + sh*Z1*#3) into the flanks of *SCID* mice (n = 7) and we obtained similar results (Figure 5C). In another SCLC cell line, H1930, the reduction of PTPRZ1 expression decreased the rate of tumor formation under the skin in *SCID* mice as compared to the cells expressing the sh*LUC* control (Figure 5D). These results provide proof that PTPRZ1 regulates tumor growth *in vivo* and has an oncogenic function in NET progression.

## Discussion

Here we demonstrate that PTPRZ1 specifically exists in human NET tissues and PTPRZ1 has an important oncogenic role in the tumor progression of SCLC in the murine xenograft model. We also found that PTPRZ1 regulates the tyrosine phosphorylation of CaM in the response to PTN in SCLC cells. Our results indicate that the putative tumor suppressor family PTPR can support tumor progression and is required for the tyrosine phosphorylation of CaM. This study supports the idea that a new signaling pathway involving PTPRZ1 could be a feasible target for treatment of cancers. The combination of our cellular and xenograft model findings advocates for the future preclinical testing of antibody therapy or small molecule inhibitors of PTPRZ1 for the treatment of NETs and SCLC.

The linkage between oncogenic PTPRZ1 function and CaM phosphorylation is still unclear. Perez-Pinera and colleagues demonstrated that phosphorylation of Anaplastic lymphoma kinase (ALK) in PTN-stimulated cells is mediated through the PTN/PTPRZ1 signaling pathway [35], indicating that ALK might phosphorylate CaM. Further experiments are needed to address the possibility of PTN mediating its effects via ALK [35] in SCLC cells, the effects of PTN deletion on tumor growth, and the mechanism of PTN/PTPRZ1 autocrine regulation in NET cells. CaM can bind up to four calcium ions, and can undergo post-translational modifications such as phosphorylation, acetylation, methylation and proteolytic cleavage, each of which can potentially modulate its actions [34]. A prior biochemical study showed that tyrosine phosphorylation increased the association of CaM with nitric oxide synthase (NOS) [36]. Because nitric oxide (NO) and NOS are ubiquitous in malignant tumors and known to exert pro-tumor effects [37,38], PTPRZ1 may regulate NO production in SCLC cells by changing the tyrosine phosphorylation status of CaM. Tumor cell-derived NO promotes tumor progression by induction of tumor-cell invasion, proliferation and the expression of angiogenic factors [37,38]. Indeed a recent research article demonstrated that glioma stem cell proliferation and tumor growth are promoted by iNOS [39].

With regards to another aspect of its oncogenic role, PTPRZ1 has a huge extracellular domain consisting of a alpha-carbonic anhydrase domain (CA), chondroitin sulfate proteoglycans (CS-PGs), and a fibronectin type-III domain (FNIII). PTPRZ1 expression is dramatically induced by hypoxic stress through HIF-2α [19], suggesting that PTPRZ1 may have an important role under hypoxic conditions. Recently, Jeong’s research group reported that CA was dramatically up-regulated in human SCLC tissues by proteomic analysis [40]. A possible speculation is that the CA domain of PTPRZ1 could have an important function for tumor progression of SCLC and further studies will be required to address this issue.

## Conclusions

We found that PTPRZ1 has an important oncogenic role in tumor progression in the murine xenograft model of SCLCs. Moreover we demonstrate that the binding of PTPRZ1 to its ligand PTN inactivates phosphatase activity, resulting in tyrosine phosphorylation of CaM in human tumors. These results indicate that a new signaling pathway involving PTPRZ1 could be a feasible target for treatment of NETs.

## Abbreviations

SCLC: Small cell lung carcinoma; PTP: Protein tyrosine phosphatase; PTPRZ1: Protein tyrosine phosphatase receptor Z1; NETs: Neuroendocrine tumors; PTN: Pleiotrophin; CaM: Calmodulin; shRNA: Small Hairpin RNA.

## Competing interests

The authors declare that they have no competing interests.

## Authors’ contributions

Histological diagnostics for pathological human tissues were carried out by GI, MK, SF, TK and AO. AO conceived the study. All experiments were optimized and performed by HM. Manuscript were written by HM and revised by YH, GI and AO. All authors have read and approved this manuscript.

## Pre-publication history

The pre-publication history for this paper can be accessed here:

http://www.biomedcentral.com/1471-2407/12/537/prepub
